# Microbial vitamin biosynthesis: Annotated selection of World Wide Web sites relevant to the topics in microbial biotechnology

**DOI:** 10.1111/1751-7915.14108

**Published:** 2022-07-01

**Authors:** Lawrence P. Wackett, McKnight Professor

**Affiliations:** ^1^ Department of Biochemistry, Molecular Biology & Biophysics, BioTechnology Institute University of Minnesota St. Paul MN USA

## Riboflavin biosynthesis

### https://www.frontiersin.org/articles/10.3389/fbioe.2020.570828/full

Riboflavin, or vitamin B2, is essential for humans to make the coenzymes FAD and FMN. It is also fed to domestic animals. This review article discusses well‐known bacteria and yeast producers of riboflavin.

## Microbial resources for cobalamin biosynthesis

### https://www.researchgate.net/publication/351136590_Microbial_and_Genetic_Resources_for_Cobalamin_Vitamin_B12_Biosynthesis_From_Ecosystems_to_Industrial_Biotechnology

This ResearchGate page provides access to a recent review article on the microbial biosynthesis of cobalamin, also known as vitamin B12.

## *E. coli* cobalamin synthesis engineering

### https://www.nature.com/articles/s41467-018-07412-6

This paper details genetic engineering of *E. coli* for cobalamin biosynthesis.

## Vitamin biosynthesis business

### https://www.sbmicrobiologia.org.br/PDF/The%20business%20of%20biotechnology.pdf

This review on the microbial production of metabolites for commercial purposes has an excellent section on vitamins.

## Vitamin A production with yeast

### https://pubs.acs.org/doi/abs/10.1021/acssynbio.9b00217

Vitamin A is a water insoluble vitamin. This paper describes its production by *Saccharomyces cerevisiae* and its extraction using an in situ water‐solvent system.

## CoQ10 biosynthesis

### https://europepmc.org/article/med/29755918

Coenzyme Q10, also known as ubiquinone, is important in energy metabolism. Microbial production methods are preferable over chemical synthesis, and several major bacterial and yeast production systems are discussed.

## Alternative biosynthetic pathways for vitamin C

### https://elifesciences.org/articles/06369

This paper describes an alternative ascorbic acid biosynthetic pathway in a photosynthetic eukaryotic cell.

## Food additives and vitamins

### https://www.princeton.edu/~ota/disk3/1984/8407/840709.PDF

This posting provides a good summary of microbial metabolite production, including the production of vitamin B2, vitamin B12, vitamin C and vitamin E.

## Process and economics for ascorbic acid

### https://link.springer.com/article/10.1007/s42452‐020‐2604‐8

This study describes a two‐step commercial plant process for producing ascorbic acid at high level and in 95% purity by the fermentation of D‐sorbitol.

## Enzyme for producing nicotinamde

### https://pubs.acs.org/doi/10.1021/op100309g

Nicotinamide is a vitamin, used in the body to produce NAD^+^. This paper describes its production via the microbial enzyme nitrile hydratase.

